# Sensitivity and Reusability of SiO_2_ NRs@ Au NPs SERS Substrate in Trace Monochlorobiphenyl Detection

**DOI:** 10.1186/s11671-015-1147-1

**Published:** 2015-11-17

**Authors:** Mengjing Hou, Yu Huang, Lingwei Ma, Zhengjun Zhang

**Affiliations:** State Key Laboratory of New Ceramics and Fine Processing, School of Materials Science and Engineering, Tsinghua University, Beijing, 100084 People’s Republic of China; Key Laboratory of Advanced Materials (MOE), School of Materials Science and Engineering, Tsinghua University, Beijing, 100084 People’s Republic of China

**Keywords:** Monochlorobiphenyl, Surface-enhanced Raman scattering, Reusability, Trace detection, Principal component analysis

## Abstract

Surface-enhanced Raman scattering (SERS) effect is quite preferred to detect trace pollutants, and reusable SERS substrate is of important practical value. In this research, a kind of effective SiO_2_ nanorods (NRs)@ Au nanoparticles (NPs) substrate was fabricated completely with physical methods, and it was quite sensitive so that 1 × 10^−6^ M monochlorobiphenyl (CB) could be detected. Furthermore, congeners of CB could be detected by reusing this kind of SERS substrate, and the cleaning treatment between every two detections was very simple. The excellent performance of the reusable SERS substrate indicated its great application potential.

## Background

Trace pollutant detection is increasingly concerning during the last several decades, on account of the danger to environment and public health caused by the accumulation of trace harmful chemicals [1–5]. The detection method based on surface-enhanced Raman scattering (SERS) effect has been preferred in recent years, [6–10] for it is accurate, rapid, and convenient. On the surface of noble metal nanostructures, localized electric field intensity would be enhanced due to the localized surface plasmon resonance (LSPR) excited by laser [11–14]. By being adsorbed on the SERS substrate, trace pollutants could be detected with a Raman spectrometer. To detect chemicals of lower concentration, a lot of effort has been taken by researchers to improve the SERS substrates. For example, kinds of Au or Ag nanostructures were synthesized with HAuCl_4_ or silver nitrate [15–18], while the ones fabricated by sputtering were also reported [19–21].

With well-designed SERS substrates, trace polychlorinated biphenyls (PCBs) were detected as reported. PCBs are a series of persistent organic pollutants which could accumulate in human bodies through the food chain rather than degrade even if their initial concentration in soil and water is low. With the least chlorine atom in the molecule, monochlorobiphenyl is highly volatile and easy to disperse throughout the environment, which leads to greater danger. As toxic chemicals, PCBs including monochlorobiphenyl could harm the reproductive system, integumentary system, brain, and so on and are also carcinogenic [22, 23]. Therefore, it is quite necessary to detect trace PCBs so that to prevent their diffusion. However, it is difficult for PCB molecules to adsorb on the surface of Au and Ag. Especially for SERS substrates fabricated with some kinds of solution, the limit of detection (LOD) is unsatisfactory. Hence, surface modification was generally carried out on the noble metal nanostructures. With β-cyclodextrin or alkanethiol, PCB molecules would be caught tightly near the metal surface, so that their Raman scattering signal would be enhanced significantly [24–26]. The modified molecules, however, would meanwhile prevent desorption of PCB molecules due to the action of van der Waals forces, which makes the reuse process of the SERS substrate quite hard, and the signal intensity of re-adsorbed PCBs becomes weaker obviously [27].

To achieve the objective of reusing SERS substrates to detect trace PCBs which is quite valuable in the actual application situation, a kind of SERS substrate fabricated with physical methods completely was designed and optimized in this research. With abundant “hot spots” formed on the rough surface of Au nanostructures and in the gaps between them, [28, 29] the SERS substrate showed excellent trace detection ability. Moreover, without other chemicals existing on the surface of Au, PCB molecules could contact the “hot spots” directly, as well as be desorbed and re-adsorbed easily. The experimental results of reusing the SERS substrate several times to detect the same type or different type of PCB congeners successfully demonstrated the effectiveness of this kind of substrate. To characterize the trace detection effect of the reusing process, the chemometrics method of principal component analysis (PCA) was employed to calculate the SERS signal intensity of the re-adsorbed PCB molecules and the desorption degree comprehensively.

## Methods

### Fabrication of SiO_2_ NRs@ Au NPs SERS Substrates

**Fig. 1:**
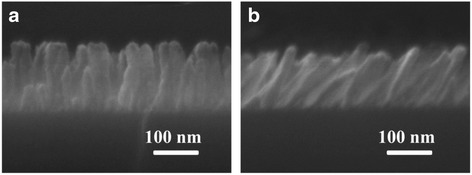
SEM morphology of **a** vertical and **b** oblique SiO_2_ nanorod array

The SiO_2_ NRs@ Au NPs SERS substrates were fabricated completely by physical vapor deposition methods. To obtain a vertical SiO_2_ nanorod array, the glancing angle deposition (GLAD) technique was employed with a DZS-500 electronic beam evaporation system (SKY Technology Development Co., Ltd. Chinese Academy of Sciences) [30]. The wafer was kept in an in-plane rotation with a certain oblique angle, so that the incident angle of the SiO_2_ beam was 86°. The height of the vertical SiO_2_ nanorods was ~140 nm, with enough space between each other as Fig. 1a shows, which ensured the Au nanoparticles could reach the side of the nanorods during the next procedure. The oblique SiO_2_ nanorod array was deposited with a method which was a little different with GLAD. By keeping the oblique plane with an angle of 86° to the horizontal plane, the wafer received more SiO_2_ nanoparticles from a certain direction; thus, the oblique nanorod array took shape. As shown in Fig. 1b, the length of the oblique SiO_2_ nanorods was ~140 nm, which was similar to the vertical nanorods.

The Au nanoparticles were sputtered on the two kinds of SiO_2_ nanorod array afterwards by a SBC-12 vacuum ion coater (KYKY Technology Co., Ltd.). Through alteration of the deposition time of Au from 60, 90, 120,…, up to 360 s, the amount and morphology of the nanoparticles were modified. Hence, 22 kinds of samples in total were prepared.

### Characterization of SERS Substrates

By scanning electron microscope (SEM, Merlin VP Compact, Carl Zeiss), the morphology of the SiO_2_ NRs@ Au NPs SERS substrates was characterized. And by transmission electron microscope (TEM, JEOL-2100F), the high-resolution image and diffraction pattern of the nanostructures were obtained.

### Preparation of Analyte Solution

To compare the SERS effect of the substrates with a different amount and morphology of Au nanoparticles on vertical as well as oblique SiO_2_ nanorod arrays, trans-1,2-bis(4-pyridyl)-ethylene (BPE) (J&K Scientific Ltd.) was employed as a probe molecule. The BPE powder was dissolved in ethanol and diluted to 1 × 10^−4^, 1 × 10^−6^, 1 × 10^−7^, 1 × 10^−8^, and 1 × 10^−9^ M, in sequence. The analytes 2-chlorobiphenyl (2-CB), 3-chlorobiphenyl (3-CB), and 4-chlorobiphenyl (4-CB) (AccuStandard Inc.) were dissolved in acetone. 2-CB and 3-CB solutions with a concentration of 1 × 10^−4^ M and 4-CB solutions with concentrations of 1 × 10^−4^, 1 × 10^−5^, 5 × 10^−6^, and 1 × 10^−6^ M were prepared.

### Adsorption of Probe Molecules

To make the molecules adsorb on the SERS substrates, proper methods were used. By immersing the SERS substrates in the solution, BPE molecules were adsorbed both physically and chemically. After being rinsed with ethanol, only chemisorbed molecules remained. Chlorobiphenyl solution was dropped on the SERS substrates, and the volumes of the droplets were 3 μL each time. To clean the adsorbed CB molecules, the SERS substrate was washed with acetone for several seconds.

### Measurements of SERS Spectra

With an optical fiber micro-Raman system (i-Raman Plus, B&W TEK Inc.), the SERS spectra of the trace chemicals were measured. The employed 785-nm laser formed a beam spot of ~85 μm in diameter on the surface of the samples, while the laser power and the integral time were adjusted according to each series of samples.

## Results and Discussion

**Fig. 2:**
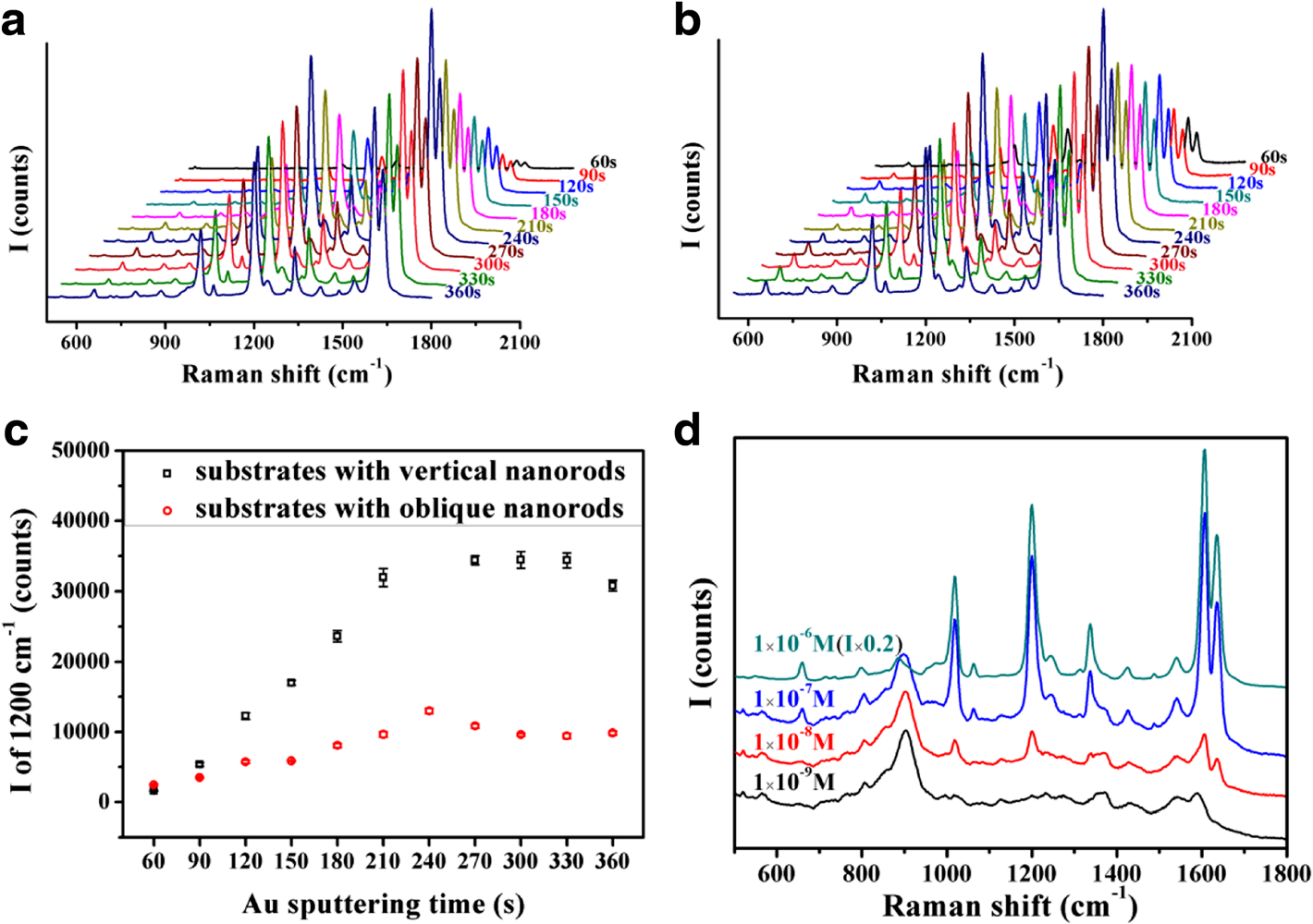
SERS spectra of 1 × 10^−4^ M BPE adsorbed on substrates of **a** vertical and **b** oblique SiO_2_ nanorods sputtered with Au nanoparticles for 60, 90,…, 360 s. **c** Intensity of characteristic Raman peak of BPE at 1200 cm^−1^ measured with all kinds of SERS substrate samples. **d** SERS spectra of 1 × 10^−6^, 1 × 10^−7^, 1 × 10^−8^, and 1 × 10^−9^ M BPE measured with the vertical SiO_2_ NRs@ Au NPs SERS substrate whose Au sputtering time is 240 s. All of the spectra were average data of spectra measured on five positions randomly selected on each SERS substrate

**Fig. 3:**
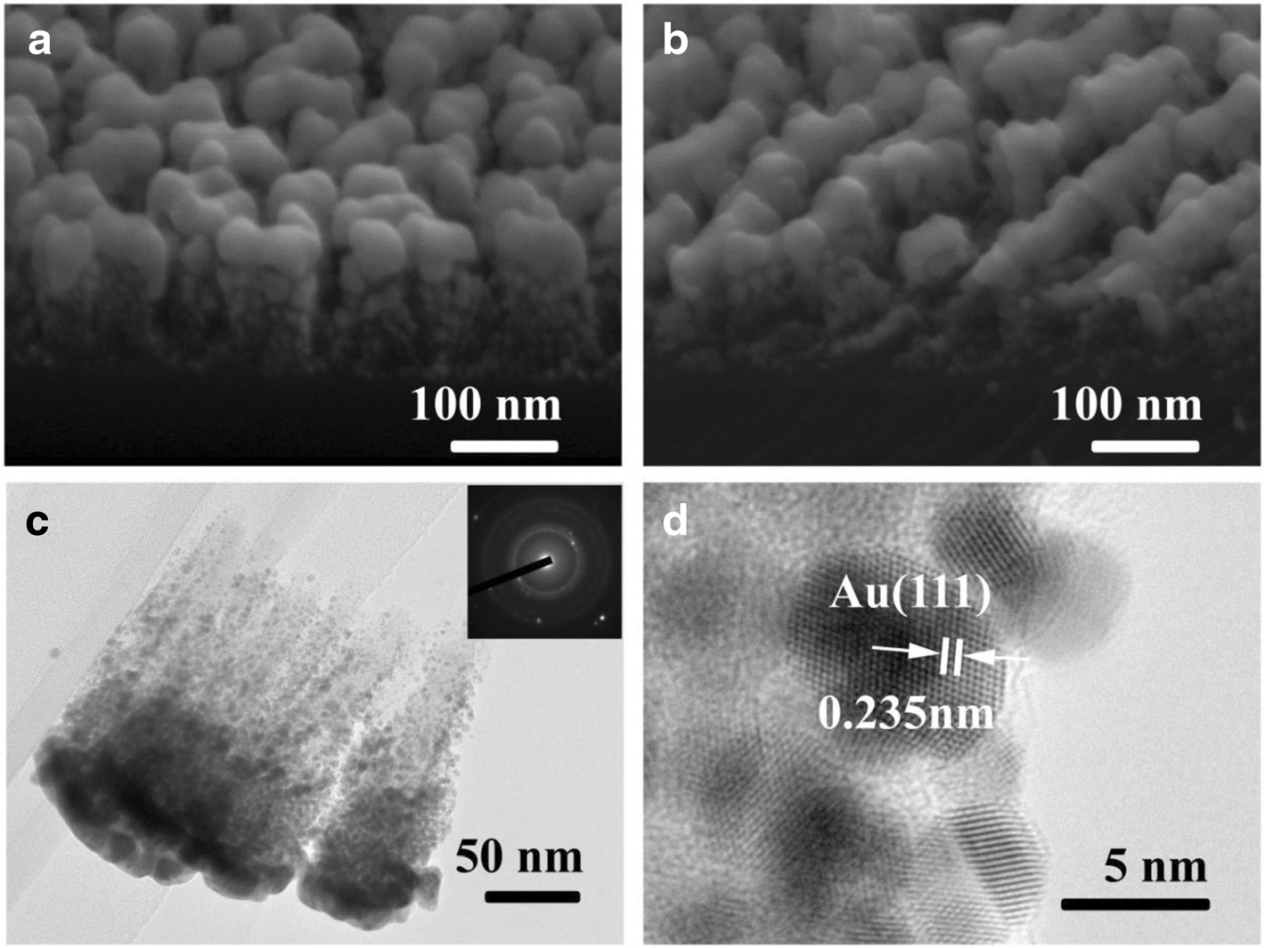
SEM morphology of SERS substrate samples fabricated by sputtering Au nanoparticles for 240 s on **a** vertical and **b** oblique SiO_2_ nanorods. **c** TEM morphology of SiO_2_ nanorods sputtered by Au for 240 s. The *inset* is the electron diffraction pattern of the sample. **d** HRTEM of Au nanoparticles decorated on SiO_2_ nanorod

The localized electric field could be significantly enhanced on the surface of the Au nanoparticles and gaps between them. On a rougher surface, the electric field intensity would be improved, and with more Au particles on the SERS substrate, the Raman signal would be stronger of course. Therefore, the Au nanoparticles used in this research were fabricated with the method of sputtering, and SiO_2_ nanorods were employed as skeletons to carry more Au nanoparticles in three-dimensional space. According to the previous studies [7], oblique Ag nanorods performed a strong SERS effect. This result leads to the idea that Au nanoparticles decorating on oblique SiO_2_ nanorods may be a good SERS substrate. Basing on this idea, vertical and oblique SiO_2_ nanorods were deposited as Fig. 1a, b showed, respectively. To obtain the substrate with the best SERS effect, the amount of Au nanoparticles adhered on the nanorods was adjusted by altering the sputtering time. A BPE solution of 1 × 10^−4^ M was employed to characterize the SERS effect of the samples with a different amount of Au nanoparticles decorated on the vertical and oblique SiO_2_ nanorods. Figure 2a b both showed the phenomenon that the SERS effect of the samples improved with the amount of adhering Au nanoparticles until the sputtering time of 240 s, and then, the Raman peaks’ intensity of BPE decreased a little when prolonging the sputtering time. The reason maybe lies in the morphology of Au nanoparticles. When too many Au nanoparticles gather on the top of SiO_2_ nanorods, they would form larger particles and prevent the incident laser from reaching the Au nanoparticles adhering on the side of the nanorods. Besides, the localized electric field would be weaker on the smoother surface of larger Au nanoparticles [31]. To compare the SERS effect of the substrates employing vertical and oblique SiO_2_ nanorods as skeletons, the intensity of the peak at 1200 cm^−1^ was marked in Fig. 2c. It could be observed that the variation laws of the SERS effect of samples employing vertical and oblique SiO_2_ nanorods as skeletons along with Au sputtering time are similar, and vertical nanorods skeletons contributed more to the SERS effect. This result’s difference with the initial guess could be explained according to the SEM photos of the substrates which were fabricated by depositing Au nanoparticles for 240 s on vertical (Fig. 3a) and oblique (Fig. 3b) SiO_2_ nanorods. Au nanoparticles adhering on the top of vertical nanorods were discrete, whereas the particles on the top of oblique nanorods formed lines which would generate a weaker localized electric field. Meanwhile, nanoparticles on the side of the oblique nanorods would be blocked so that their SERS effect could not work. Though the oblique SiO_2_ nanorods provided a larger surface to load Au nanoparticles, they were not suitable to act as skeletons on SERS substrates.

The best SiO_2_ NRs@ Au NPs SERS substrate fabricated by sputtering Au for 240 s on vertical nanorods was measured with BPE solution. On the SERS spectra of BPE in the concentrations of 1 × 10^−6^, 1 × 10^−7^, 1 × 10^−8^, and 1 × 10^−9^ M in Fig. 2c, the characteristic peaks could be recognized. The LOD for BPE could reach 1 × 10^−9^ M, and the enhancement factor was estimated as about 10^8^, which demonstrated the optimized SERS substrate was quite effective. The effectiveness should be attributed to abundant Au nanoparticles adhering on the whole nanorods, as the TEM photo (Fig. 3c) and HRTEM photo (Fig. 3d) showed.

**Fig. 4:**
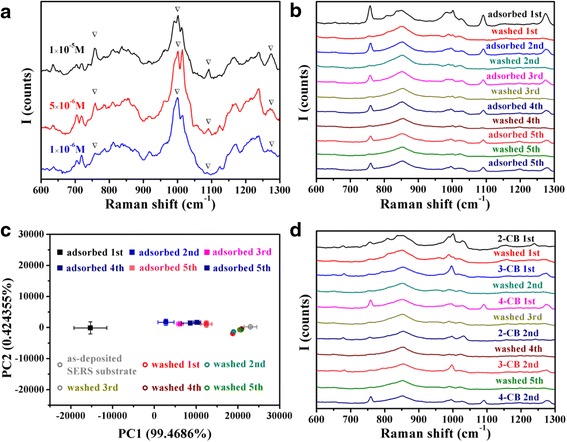
**a** SERS spectra of 1 × 10^−5^, 5 × 10^−5^, and 1 × 10^−6^ M 4-CB measured with the substrate fabricated by sputtering Au for 240 s on SiO_2_ nanorods. **b** SERS spectra of 1 × 10^−4^ M 4-CB repeatedly detected and washed. **c** PCA score plot of SERS spectra of 4-CB repeatedly detected and washed. **d** SERS spectra of 1 × 10^−4^ M 2-CB, 3-CB, and 4-CB repeatedly detected and washed. All of the spectra were average data of spectra measured on three positions randomly selected on each SERS substrate

The optimized SiO_2_@Au SERS substrate was then applied to detect trace monochlorobiphenyl. Firstly, SERS spectra of 1 × 10^−5^, 5 × 10^−6^, and 1 × 10^−6^ M 4-CB were measured. In Fig. 4a, characteristic peaks at 760, 1000, 1090, and 1274 cm^−1^ were assigned to ring deformation, ring trigonal breathing, C-Cl stretching, and C-C bridge-stretching mode, respectively [32, 33], could be observed. The LOD of 1 × 10^−6^ M obtained without the assistance of surface modification was actually satisfactory [32, 34].

Moreover, the SiO_2_@Au SERS substrate was used repeatedly to detect trace monochlorobiphenyl. The characteristic peaks of 4-CB detected with an as-deposited SERS substrate in Fig. 4b were significantly stronger than the ones detected after the SERS substrate washed by acetone. However, it is nice to see that the Raman signal intensity kept nearly constant for the following several times of re-detection. Besides, there was almost no characteristic peaks of 4-CB after the SERS substrate was washed, which showed that the substrate could be cleaned easily, benefiting from the weak forces between the CB molecules and the Au nanoparticles rather than the strong van der Waals force caused by surface modification molecules. With the chemometrics method of PCA, the reusability of the SERS substrate was analyzed quantitatively. Employing the spectra of 4-CB measured at the first time and the as-deposited SERS substrate as the calibration data, a PCA model was established and the score plot of principal component 1 (PC1) and PC2 was exhibited in Fig. 4c. Judging from the variance contribution, PC1 has already reflected most information of the spectra differences. Therefore, the reusing effect could be represented by the rate of PC1 score change which should be calculated through dividing the PC1 score difference between the re-detected and the as-deposited substrate spectra by the difference between the firstly detected and the as-deposited substrate spectra. As Table 1 shows, the 4-CB re-detection and cleaning effect of the SERS substrate are both satisfactory.

**Table 1:** Rate of PC1 score change of 4-CB re-detection spectra and acetone-washed SERS substrate spectra

4-CB detection order	2nd time	3rd time	4th time	5th time	6th time
Rate of PC1 score change (%)	52.78	44.00	37.55	27.50	33.25
Acetone-washing order	1st time	2nd time	3rd time	4th time	5th time
Rate of PC1 score change (%)	11.02	10.47	6.65	5.52	5.82

Furthermore, the SERS substrate was applied to re-detect different congeners of monochlorobiphenyl. Observing from Fig. 4d, CB molecules could be washed away nearly completely, and other kinds of CB molecules could be detected even if the SERS substrate had been used several times. These spectra indicated that analytes of different chemical structures could also be detected with the reused substrate, without interference by other kinds of analytes, which widened the application range of the reusable SERS substrate.

## Conclusions

In this research, the SiO_2_ NRs@ Au NPs SERS substrate was designed and optimized by adjusting the morphology of nanorods and nanoparticles. Resultingly, the SERS substrate performed an enhancement factor of about 10^8^, and its LOD for 4-CB could reach 1 × 10^−6^ M, due to the great amount of “hot spots” and its directly contacting analytes. This kind of SERS substrate fabricated completely with physical methods could be reused to detect trace CB molecules of the same or different chemical structures, through easy cleaning treatments, because of its weak adsorption for CB compared to the stronger interaction existing on the surface modified substrates. By PCA method, the re-detection and cleaning effect of the SERS substrate was characterized quantitatively, and the performance of the substrate was quite excellent.
